# Kinetic Study of Encapsulated β-Carotene Degradation in Aqueous Environments: A Review

**DOI:** 10.3390/foods11030317

**Published:** 2022-01-24

**Authors:** Vera Lavelli, Jolanta Sereikaitė

**Affiliations:** 1 Department of Food, Environmental and Nutritional Sciences (DeFENS), University of Milan, 20133 Milan, Italy; 2 Department of Chemistry and Bioengineering, Vilnius Gediminas Technical University, 10223 Vilnius, Lithuania; jolanta.sereikaite@vgtu.lt

**Keywords:** β-carotene, encapsulation, emulsion, liposomes, molecular complexes

## Abstract

The provitamin A activity of β-carotene is of primary interest to address one of the world’s major malnutrition concerns. β carotene is a fat-soluble compound and its bioavailability from natural sources is very poor. Hence, studies have been focused on the development of specific core/shell micro- or nano-structures that encapsulate β-carotene in order to allow its dispersion in liquid systems and improve its bioavailability. One key objective when developing these structures is also to accomplish β-carotene stability. The aim of this review is to collect kinetic data (rate constants, activation energy) on the degradation of encapsulated β-carotene in order to derive knowledge on the possibility for these systems to be scaled-up to the industrial production of functional foods. Results showed that most of the nano- and micro-structures designed for β-carotene encapsulation and dispersion in the water phase provide better protection with respect to a natural matrix, such as carrot juice, increasing the β-carotene half-life from about 30 d to more than 100 d at room temperature. One promising approach to increase β-carotene stability was found to be the use of wall material, surfactants, or co-encapsulated compounds with antioxidant activity. Moreover, a successful approach was the design of structures, where the core is partially or fully solidified; alternatively, either the core or the interface or the outer phase are gelled. The data collected could serve as a basis for the rational design of structures for β-carotene encapsulation, where new ingredients, especially the extraordinary natural array of hydrocolloids, are applied.

## 1. Introduction

Vitamin A deficiency, which is defined as having serum retinol below 300 ng/mL, is a major malnutrition concern. Physiological needs for vitamin A are particularly relevant for young individuals and pregnant women, which represent the groups of the population at the highest risk of deficiency [1,2,3]. Vitamin A is found in foods from animal origin, the highest levels being in organ meats (liver, kidney), and to lesser extent in other meat products, milk and milk products. Moreover, compounds with provitamin A activity (carotenoids), such as β carotene, are found in yellow and orange fruits and vegetables [4,5].

A scientific consensus was reached on the provitamin A function of β-carotene and the role of β-carotene in the accomplishment of the recommended total vitamin A intake [6]. Hence, provitamin A carotenoid-rich products (especially carrots, palm oil, gac oil, buriti, mango, sweet potato, apricot, and green vegetables) have received attention for combating vitamin A deficiency [7]. The role that β-carotene could play as a source of vitamin A is relevant, since an integrated modelling study has revealed that replacing animal-source foods with plant-based sources is particularly effective at addressing environmental, food security and public health objectives [8]. Moreover, since the principles of the circular economy call for the transition to more sustainable production systems [9], relevant opportunities arise from recovering β-carotene from various by-products of food processing, including carrot, apricot and mango waste [10].

However, vitamin A deficiency is not only related to low dietary intake, but also to its poor bioavailability, and especially to the poor bioavailability of carotenoids from vegetable sources [11,12]. In vitro studies have been performed to obtain insights on the bioaccessibility of fat-soluble vitamins and provitamins, i.e., their release from the lipid phase of the food matrix and solubilization within the mixed micelles formed from lipid digestion products, which is the process governing their bioavailability [13,14]. Through in vitro digestion studies, it was found that the bioaccessibility of β-carotene in raw carrot puree is very low (3.8%), and it increases only moderately after thermal treatments of oil-added purees (10.7%) [15]. To optimize the use of β-carotene, an approach is the extraction of this compound from food sources, such as carrots, and then its encapsulation into specific nano- or micro-structures that enable its homogeneous distribution in the food matrix and improve its bioaccessibility [14]. Extraction is commonly achieved with organic solvents such as hexane, acetone, methanol and ethanol, as well as various solvent combinations, but more sustainable approaches have been developed using microwave-assisted extraction [16,17], ultrasound-assisted extraction [18], supercritical fluid extraction [19,20] and pressurized liquid extraction [21,22]. β-carotene was also extracted from carrot peel waste using a green procedure without any solvent, based on water-induced hydrocolloidal complexation with pectin [23]. After extraction from the natural source, β-carotene encapsulation is obtained in aqueous environments. To this aim, water-soluble molecular complexes with protein or polysaccharides are produced, in which the biopolymers can auto-assemble, forming a hydrophobic cavity that can hold β-carotene as a guest molecule. Alternatively, various types of emulsions are obtained in which the hydrophobic β-carotene is dissolved in a lipid phase that is separated by the water environment through an amphiphilic interface [24,25,26]. All these micro- and nano-structures could then be dissolved into the water fraction of foods.

Previous review studies have focused on the technology for the microencapsulation [24] and nanoencapsulation of carotenoids [25,26], and the resultant positive effects on bioaccessibility were illustrated. Conversely, data on the stability of encapsulated β-carotene are scattered, while a comparison of different encapsulation approaches through kinetic data can generate new information on the interactions among β-carotene, co-encapsulated compounds, host molecules or carrier lipid phases and water/oil interfaces. In fact, another crucial point is to ensure β-carotene stability, since due to its many conjugated double bonds, β-carotene is particularly prone to chemical degradation during food processing and storage, mainly depending on oxygen, light exposure, temperature and time [27,28,29,30,31]. The pathway for the autoxidation of β-carotene was proposed to begin with a cis−trans isomerization that may take place at C9–10 or C13–14 or C15–15′; then, the rupture of the cis double bond occurs with the creation of a diradical molecule with an oxygen atom bound to just one carbon atom; the next step was hypothesized to be the generation of a peroxide intramolecular cycle that triggers the formation of apocarotenoids upon its rupture [27]. For pure β-carotene, a first-order kinetics was found for the global process [32].

The aim of this review study is, therefore, to collect kinetic data on β-carotene degradation (rate constants, activation energy) and how it is affected by the encapsulation system, and to derive knowledge on the technological approaches to increase β-carotene stability in aqueous environments. The choice of selecting a pure carotenoid compound instead of a natural carotenoid-rich extract allowed us to understand the behavior of the different compounds used for micro- and nano-structure formation. As a comparison, kinetic data on β-carotene degradation in natural sources of β-carotene, such as carrot-based products, are also reported.

## 2. Methods

### 2.1. Literature Study

The literature study was performed using two databases (Scopus and Web of Science), considering papers from 2016 to the end of 2021. Firstly, the search included the following keywords: “carotene” and “kinetic”. Papers reporting the development of an encapsulation technology for β-carotene and the kinetic study of its stability were selected. Other relevant papers were found among those cited in the papers published from 2016 to 2021. In order to compare the kinetic data obtained by various authors, the stability of the system under dark conditions was only considered, since the effect of light exposure was reported only in a few studies, with different lighting conditions applied, thus excluding the opportunity of comparison.

### 2.2. Modelling

If not indicated by the original paper, the half-life of β-carotene was interpolated using the graphs in the study reporting the time course of degradation during storage. Alternatively, when a first-order kinetic for β-carotene degradation was modelled, the half-life was calculated as:

t_1/2_ = 0.693/k (1)

where k is the rate constant at the temperature considered [33].

To calculate the activation energy, if not reported in the original paper, the Arrhenius equation was used, as follows:

ln(k_1_/k_2_) = (1/T_2_ − 1/T_1_) × Ea/R (2)

where k_1_ and k_2_ are the rate constants at the temperatures T_1_ and T_2_ (Kelvin), respectively, Ea is the activation energy (J/mol) and R is the universal gas constant (8.314 J × K^−1^ × mol^−1^) [33].

## 3. Results

### 3.1. Rate of β-Carotene Degradation in Carrot Juice and Puree

Carrot processing into juices and purees causes both heating and shear stress that might affect β-carotene content due to a combination of different and contrasting effects. Processing causes the inactivation of peroxidase activity, which is involved in the enzymatic oxidation of β-carotene. Nevertheless, processing causes the degradation of cell structures, thus increasing the exposure of carotenoids to oxygen, which promotes autooxidation, and decreases the content of ascorbic acid, which can provide protection to β-carotene [34]. Conventional blanching treatment (100 °C for up to 8 min) and vacuum-steam pulsed blanching with a steam application for up to eight cycles, each one lasting for 20 s at 20 kPa and 110 kPa, increased β-carotene content with respect to that of fresh carrot, most probably due to enzyme inactivation [35]. According to the European Chilled Food Federation, heat treatment processes equivalent to F90 °C = 10 min for the 6-D inactivation of nonproteolytic *Clostridium botulinum* can provide a shelf life of up to 42 d at 5 °C [36]. Using the kinetic data previously found for pure β-carotene, i.e., activation energy of 39 kJ/mol and a rate constant at 50 °C (k_50°C_) of 0.001 min^−1^ [32], a theoretical loss of pure β-carotene upon heat treatment at 90 °C for 10 min would be moderate, equal to 5%. However, the stability of β-carotene decreased when prolonging the holding time at refrigerated or room temperatures, i.e., during the storage phase of pasteurized carrot juice, with a half-life of 33 d at 4 °C (k_4°C_ = 0.021 d^−1^, Table 1) [34]. As an alternative to thermal treatments, non-thermal technologies for carrot juice and puree preservation are emerging to decrease the energy input. Carrot juice was pasteurized via a high intensity pulsed electric field treatment consisting of bipolar square wave pulses of 6 μs at 200 Hz using 35 kV cm^−1^ for 1500 μs, with an electric energy density input of 8269 kJ × L^−1^. Nevertheless, β-carotene degradation during storage occurred with a half-life for β-carotene of 39 d at 4 °C (k_4°C_ = 0.018 d^−1^, Table 1) [34]. The high pressure treatment of carrot juice, at up to 600 MPa for 5 min at 22 °C, resulted in the high degradation of carotenoids due to insufficient enzyme deactivation [37]. In another approach, carrot juice was submitted to high pressure treatment at different pressures (60 MPa, 120 MPa and 180 MPa), passes (one and three passes) and inlet temperatures (25 °C and 60 °C). The highest retention during storage was found for the juice treated at 180 MPa, one pass and 60 °C, with a β-carotene half-life of 39 d at 4 °C (k_4°C_ = 0.018 d^−1^, Table 1) [38]. Based on the kinetics so far described, and =considering that a long shelf-life is advisable from the perspective of the circular economy, the storage phase resulted to be the most relevant for β-carotene degradation.

### 3.2. Rate of Encapsulated β-Carotene Degradation in Water-Soluble Molecular Complexes

Molecular complexes with polysaccharides or proteins have been designed to encapsulate β-carotene, improving its stability and water solubility. Differing from most of the other encapsulation systems, for which the basic architecture is an emulsion [39], molecular complexes do not require an oil phase (Table 2). These structures can be affected by physical instability because they tend to associate with each other due to attractive forces [40]. Factors affecting the stability of protein or polysaccharide complexes are the size and the surface charge, as observed for the emulsions [39,40,41]. In fact, a small particle size (d < 200 nm) creates a high level of stability to gravitational separation due to the dominant Brownian forces. Additionally, the zeta potential (ζ-potential) is related to stability because levels close to zero promote aggregation due to the absence of charge repulsions [39,40,41]. Indeed, the stability of these encapsulation systems is dependent on the pH and ionic strength, as these factors change the ζ-potential. Molecular complexes were prepared using the mechanochemical methods, i.e., kneading, freeze drying or sonication using β-carotene dissolved in ethanol and chitooligosaccharides obtained via the acid hydrolysis of chitosan. In this system, the carrier itself had biological activity, thus providing additional functionality. The highest encapsulation efficiency, i.e., 78%, was found for complexes prepared via sonication, which had a mean hydrodynamic diameter of 79 nm and a ζ-potential of 16.5 mV [42]. This approach increased carotene stability at high temperatures (up to 100 °C for 30 min) and over a wide pH range (3–8). The half-life of β-carotene encapsulated in chitooligosaccharides via sonication was 28 d at 24 °C with a pH of 7.0 (k_24°C_ = 0.024 d^−1^, Table 1) [42,43].

Zein nanocapsules represent another strategy for the encapsulation of hydrophobic bioactive compounds. In fact, due to its inherent hydrophobicity, zein can self-assemble after mixing with an anti-solvent [44]. The conjugation of zein with polysaccharides, such as the positively charged carboxymethyl chitosan, was proposed to enhance the physical stability of nanocapsules [45]. In line with this strategy, β-carotene was encapsulated via anti-solvent precipitation with zein alone, a binary system made of zein and carboxymethyl chitosan, and a ternary system made with zein, carboxymethyl chitosan, and tea polyphenols [40]. Tea polyphenols were added to increase both chemical and physical stability because they possess abundant hydroxyl groups that exhibit antioxidant activity and have the ability to form hydrogen bonds with proteins and polysaccharides. Compared with zein unitary and zein–carboxymethyl chitosan binary complexes, it was interesting to note that ternary complexes had a lower particle size, i.e., approximately 80 nm and a higher surface charge, i.e., approximately 40 mV. However, the encapsulation efficiency was not reported. The ternary system resulted in higher stability during storage. In fact, the half-life for β-carotene degradation at 20 °C was 10, 14, and 38 d (k_20°C_ = 0.068, 0.048, and 0.018 d^−1^, Table 1) for the zein, zein and carboxymethyl chitosan, and zein, carboxymethyl chitosan, and tea polyphenol systems, respectively [40]. The stability of these systems was investigated in the temperature range of 4–60 °C and, hence, the activation energy for β-carotene degradation could be calculated using the Arrhenius equation, resulting in values in the range of 7–17 kJ/mol, depending on the presence of carboxymethyl chitosan and tea polyphenols (Table 3). This relatively low value indicates the moderate effect of temperature fluctuation on encapsulated β-carotene degradation.

### 3.3. Rate of β-Carotene Degradation in Liposomes Dispersed in Water

Liposomes are vesicles with an aqueous core surrounded by amphiphilic polar lipids, generally phospholipids, which produce a bilayer structure. Hydrophobic substances can be entrapped within the hydrophobic bilayer [46] (Table 2). A small radius of curvature imposes constraints on the phospholipid molecules, disrupting the regular packing of the vesicles and, hence, generating instability [47]. For these encapsulation system, the main instability processes are flocculation, coalescence, and creaming (or sedimentation), which ultimately lead to the complete separation of the two constituting phases [47]. Hence, the physical stability of these systems depends on the particle size and surface charge [39], as discussed above for molecular complexes. One of the key parameters for the liposome structure is the transition temperature, where the liposomes lose their ordered packing structure due to the melting of the hydrocarbon chains, i.e., they undergo a phase transition from gel to liquid crystalline. Since natural phospholipids contain hydrocarbon chains that differ in length, phase transition occurs over a wide temperature range. Cholesterol is generally added to the phospholipid structure at over 20% because it decreases the transition temperature and increases stability [47]. β-Carotene was encapsulated in liposomes either alone or with ascorbic acid via the ethanol injection method using yolk lecithin, cholesterol, and a phosphate buffer solution (pH 6.8), with an encapsulation efficiency of 98%. The co-encapsulation of ascorbic acid and β-carotene could significantly improve the storage stability of β-carotene, with a half-life at 25°C increasing from 6 d in the absence of ascorbic acid to more than 30 d in the presence of ascorbic acid (k_25°C_ not reported, Table 1) [48].

### 3.4. Rate of Encapsulated β-Carotene Degradation in Oil-in-Water (O/W) and Oil-in-Gel (O/gel) Emulsions

O/W emulsions are structures with a lipophilic core where a lipophilic bioactive compound is entrapped, with a hydrophilic shell (aqueous phase) and an amphiphilic interface consisting of the emulsifier [39] (Table 2). They can undergo flocculation, coalescence, and creaming (or sedimentation) that ultimately lead to the complete separation of the two constituting phases, and the rate of these processes depends on particle size and surface charge [39]. Emulsions for β-carotene delivery were prepared via high shear homogenization using medium chain triglyceride oil and Arabic gum as an emulsifier. Moreover, since β-carotene is degraded through free radical-mediated autoxidation reactions, antioxidants that act as free radical scavengers, such as ascorbyl palmitate and α-tocopherol, were added to the emulsion [46]. Minor differences due to antioxidant addition were found in the particle size and in the ζ-potential of the emulsion, which were approximately 600 nm and −25 mV, respectively, while the encapsulation efficiency was not reported. As expected, both ascorbyl palmitate and α-tocopherol increased the stability of β-carotene. Indeed, the half-life for β-carotene degradation varied from 7.2 d to 50 d at 25 ° C (k_25°C_ = 0.0955 and 0.0139 d^−1^, Table 1) in the absence or presence of antioxidants, respectively [49]. The stability of β-carotene in these systems was investigated in the temperature range of 4–65 °C, and the activation energy for degradation was found to be similar both in the absence and in the presence of ascorbyl palmitate, i.e., 49.8 and 45.7 kJ/mol, respectively (Table 3) [49]. These values were higher than the activation energy calculated for the molecular complexes of β-carotene with zein, indicating a greater sensibility to temperature fluctuations.

One promising method of emulsifying bioactive compounds is the sequential adsorption of polyelectrolytes, called the layer-by-layer technique. The multilayer emulsion is formed by coating the oil droplets with a protein layer, followed by polysaccharides with an opposite charge (Table 2). These structures have been designed to increase stability towards environmental triggers, such as pH, salt concentration, and temperature [50,51]. The emulsion interface also affects oxygen transport, as the oxygen diffusion coefficient at 20 °C is 2.2 × 10^−9^ m^2^/s in water, and it decreases to 0.14 × 10^−16^ m^2^/s in whey protein-based emulsion [52]. On the other hand, data on oxygen diffusion coefficients for multiple-layer emulsions with various thicknesses are not available. An oil phase made of medium-chain triglycerides and β-carotene was homogenized with a whey protein solution and then submitted to microfluidification. Following this step, flaxseed gum was slowly added to the O/W emulsion. The resulting oil droplet size and a ζ-potential increased with increasing flaxseed gum content up to 400 nm and −15 mV, respectively [53]. The retention of β-carotene was studied upon storage at 55 °C, which demonstrated an increased stability in the layer-by-layer emulsion with respect to the single emulsion, with a half-life of 6 and 3 d, respectively (k_55°C_ not reported, Table 1) [53].

Another proposed approach was to functionalize the emulsifier via chemical modification in order to improve its emulsifying properties and/or its barrier properties. However, this approach sometimes led to scarce results in terms of β-carotene protection. To this aim, the bacterial β-glucan called curdlan was conjugated with ferulic acid. This biopolymer–polyphenol conjugate had increased radical scavenging activity, emulsifying ability, and stability against flocculation and coalescence with respect to the native curdlan [54]. O/W emulsion with an oil droplet size of 3.4 µm and ξ-potential of −30 mV were obtained via the high pressure homogenization of β-carotene dissolved in corn oil and the functionalized polysaccharide solution. However, even if the degradation of encapsulated β-carotene was slower with respect to that of free β-carotene, the stability was low, with a half-life of 7 d at 25 °C (k_25°C_ not reported, Table 1) [55]. The functionalization of tartary buckwheat bran, which is rich in proteins, was performed by both noncovalent and covalent linkage with rutin, and the complexes were used to fabricate nanoemulsions of β-carotene dissolved in sunflower oil via high-pressure homogenization. The covalent complex exhibited higher emulsifying performance, and the corresponding nanoemulsion had a lower droplet size, i.e., 243.4 nm, and a higher absolute value of the ζ-potential, i.e., −32.7 mV, compared to the noncovalent complex and to the complex without rutin. Moreover, the covalent complex displayed improved stability of β-carotene during storage at 55 °C, with a half-life higher than 28 d, while the noncovalent complex had a half-life of 28 d, and the complex without rutin had a half-life of 7 d only (k_55°C_ not reported, Table 1) [56]. The functionalization of low methoxy pectin was also achieved via covalent complexation with soy peptide, corn peptide, or whey protein peptide. Then, O/W emulsions were prepared to encapsulate β-carotene dissolved in camellia oil, using the functionalized pectins as emulsifying agents by mixing them via high-speed shear emulsification. The droplet particle size was about 21 µm in the absence of peptides, and decreased to about 18, 14, and 11 µm in the presence of corn peptide, whey peptide, and soy peptide, respectively, while the ζ-potential was approximately −20 mV for all the systems. The half-life of β-carotene in the pectin emulsions was only 4 d at 4 °C, while it was higher than 28 d for the whey peptide-conjugated pectin and the soy-conjugated pectin (k_4°C_ not reported, Table 1). This result was attributed to the formation of thicker interfacial layers in the protein–polysaccharide conjugates. On the other hand, the pectin–corn peptide conjugate was not effective at increasing β-carotene stability [57]. Another excellent emulsifier, ovalbumin, was conjugated with dextran via two methods, i.e., wet heating and microwave heating. The ζ-potential of obtained droplets was similar and highly negative (~−30 mV) at neutral and alkaline pH values. The droplet size of β-carotene emulsions stabilized using ovalbumin–dextran emulsion was larger than that of the ovalbumin emulsion, and this finding was attributed to: (i) the larger molecule weights of ovalbumin–dextran conjugates compared with ovalbumin, and (ii) the thick interfacial layers of the ovalbumin–dextran conjugates with respect to ovalbumin. Under thermal treatment at 90 °C for 30 min, the β-carotene retention of the ovalbumin–dextran emulsions obtained by microwave heating and the ovalbumin–dextran emulsions obtained by wet heating were 55.55% and 50.25%, compared to 44.7% for ovalbumin-stabilized ones, while the stability during storage was not reported [58].

The modification of the physical status of the continuous phase was also proposed to increase β-carotene stability. In particular, oil-in-gel (O/gel) systems were designed to substitute a liquid phase into a gelled phase in order to increase physical stability. Moreover, chemical stability is expected to increase, since oxygen transport is affected by the viscosity of the medium (Table 2). Indeed, it was reported that the oxygen diffusion rate is 2.48 × 10^−9^ m^2^/s in water, while it decreases to 1.06 × 10^−9^ m^2^/s in Miglyol, a model viscous liquid oil [59]. Hence, the gelation of the continuous phase, which is a feature of the O/gel systems, was applied to decrease the rate of oxygen diffusion at the air/continuous phase interface and at the continuous phase/oil droplet interface. One structuring agent is sodium alginate, which can interact with Ca2+, leading to the formation of complexes that induce the spatial molecular rearrangement of the backbone chain known as egg box structure conformation [60]. An O/gel system was designed by using β-carotene dissolved in canola oil, with Tween 80 as an emulsifier and alginate-Ca gel as the continuous phase, in comparison with an O/W system with alginate in a water continuous phase [61]. The oil droplets obtained were in the sub-micron region (0.22 µm < Sauter diameter < 0.89 µm). However, the oil droplet size was higher in the system where alginate gelation occurred, indicating the occurrence of lipid droplet coalescence during the gelation process. As expected, the half-life for β-carotene degradation during storage at 25 °C increased from 48 to 99 d when the alginate present in the continuous phase was gelled (k_25°C_ not reported, Table 1). In this context, the ionotropically induced crosslinking of alginate in the continuous aqueous phase resulted in the reduction of the oxygen molecular diffusivity via two mechanisms: first, the increased macroviscosity of the bulk aqueous phase due to the supramolecular junction of the egg box-ordered structures and second, the decreased specific surface area of the lipid droplets (due to the larger Sauter diameter) [61]. However, the gelling of the outer phase affected only slightly the activation energy for β-carotene degradation in the temperature range of 4–37 °C, which changed from 51.1 to 47.6 kJ/mol (Table 3) [61]. For O/gel system design, potato starch in its gelatinized or non-gelatinized forms can be used as a structuring agent together with sodium alginate. Under accelerated storage conditions (65 °C for 6 days), the retention of β-carotene was approximately 25% [62]. k-carrageenan is another structuring agent proposed to encapsulate β-carotene in O/gel via ion-mediated gelation using K+, Na+, and Ca2+ [63,64]. In fact, k-carrageenan undergoes a coil–helix conformational transition, which is accompanied by aggregative interactions between the ordered molecules, leading to the formation of a biopolymeric network [65]. To this purpose, the lipid phase was prepared by dispersing β-carotene into canola oil, which was blended with Tween 80 and then added to water and sonicated. k-carrageenan solutions were prepared by dispersing the biopolymer into water along with the counterion (Na+, K+, and Ca2+) at 90 °C. To obtain the O/gel emulsions, the β-carotene O/W emulsions were heated to 60 °C and subsequently mixed with the hot k-carrageenan. Finally, the β-carotene-loaded k-carrageenan O/W emulsions were rapidly cooled to allow sol–gel transitions. The mean lipid droplet diameter was ca. 73 nm in the absence of the counterions, while in the presence of counterions, it increased as follows: K+ (80–94 nm) < Na+ (91–106 nm) < Ca2+ (128–134 nm). The O/gel emulsions containing monovalent ions exerted the highest β-carotene retention throughout isothermal storage, particularly at high (37 and 55 °C) temperatures (k_37°C_ and k_55°C_ not reported) [66].

### 3.5. Rate of Encapsulated β-Carotene Degradation in Solid Lipid (SL) Nanoparticles and in Nanostructured Lipid (NL) Carriers Dispersed in Water

Lipid nanoparticles are emulsions with a core made of either fully or partially solidified fat, entrapping a lipophilic bioactive compound, which are dispersed in an aqueous phase by an emulsifier [67,68,69] (Table 2). These systems have been designed to increase the stability of the encapsulated compounds because the solid lipid state may reduce the diffusion processes between the aqueous phase and the lipid core. Indeed, as indicated above, the oxygen diffusion rate is 2.48 × 10^−9^ m^2^/s in water, 1.06 × 10^−9^ m^2^/s in a model viscous liquid oil, and 0.22 × 10^−9^ m^2^/s in copra oil, a model solid fat [59]. Solid liquid (SL) nanoparticles have a fully solidified core. However, the production of SL nanoparticles for β-carotene encapsulation did not always result in increased stability with respect to the emulsion having a liquid core. In fact, in one study, microstructures for β-carotene encapsulation with the same composition but different physical state of the core were obtained using hydrogenated palm kernel oil, which can be prepared both in a liquid (to form O/W emulsions) and solid state (to form SL nanoparticles) due to the hysteresis of the melting and crystallization phases. These structures were prepared via high share homogenization using whey protein isolate as an emulsifier (the encapsulation efficiency was not reported). The surface-weighted mean diameter was about 0.5 µm for both liquid O/W emulsions and SL nanoparticles, while the surface charge was not reported. Upon storage for 28 d at 15 °C, it was observed that the liquid core provided more than 90% retention of β-carotene, while the solid core provided about 80% retention. The lower stability of β-carotene in the SL nanoparticles was attributed to the crystallization of the core, which caused β-carotene expulsion. However, the conformation of lipid crystals was not provided [70].

Insights into the effects of the nature of the solid fat on the stability of bioactive compounds encapsulated in SL nanoparticles were obtained by analyzing the conformation of the crystalline structure. In fact, triglycerides with compacted crystalline conformations provide reduced space to accommodate the bioactive compounds in the lipid core, thus causing their expulsion and resulting in fast degradation [71,72]. Generally, the crystallization of saturated triglycerides occurs in the α-subcell crystals that are metastable and, hence, tend to transform into β’ and β-subcell crystals during storage [71,72]. The polymorphic transitions of the crystalline lipid α ↦ β’ ↦ β structures result in a conformational modification of the SL nanoparticles. In fact, the α-subcell crystals are spherical and provide enough place for the accommodation of β-carotene, which is highly hydrophobic and has a high melting temperature (180 °C) [71,72]. Conversely, β-subcell crystals have a platelet-like structure, which increases their surface area and induces the aggregation of SL nanoparticles due to hydrophobic interactions, thus causing β-carotene expulsion from the lipid crystals [71]. One aspect to notice is that the surfactant itself affects the core structure of SL nanoparticles. In particular, the effects of both the physical state of the carrier lipid and the type of surfactant were investigated by using either a high melting point lipid (tripalmitin) or a low melting point lipid (medium-chain triglycerides) to form SL nanoparticles or liquid O/W emulsions, respectively. High melting point lecithin or low melting point lecithin were used as surfactants, with taurodeoxycholate as a cosurfactant. These structures were formed by using a microfluidizer and resulted in a surface-weighted mean diameter of about 0.2 and 0.16 for those with a solid and liquid core, respectively, but the encapsulation efficiency was not provided [71]. When low melting point (“liquid”) lecithin was used to stabilize particles, the liquid core provided better stability with respect to the solid core, with a β-carotene degradation of 21% and 97%, respectively, after 21 d at 20 °C (k_21°C_ not reported). Indeed, the liquid emulsifier allowed a fast recrystallization of the solid core (tripalmitin) from the α- to the more stable and more compact β-subcell crystals, causing β-carotene expulsion from the crystals. Obviously, the liquid droplets remained spherical, and β-carotene was not expelled from their surface. Interestingly, when the high melting point lecithin was used, the liquid core provided lower stability with respect to the solid core, with a β-carotene degradation of 16% and 11%, respectively, after 21 d at 20 °C (k_21°C_ not reported). The high melting point lecithin retarded the recrystallization of the α-subcell crystals and, hence, β-carotene stability was improved [71]. The importance of the crystalline structure of the core of SL nanoparticles for β-carotene stability and the role of the emulsifier on the crystallization process was further confirmed by a following study in which tristearin was used as a solid lipid core and Quillaja saponin, alone or combined with high or low melting point lecithin, was used as a surfactant. The SL nanoparticles were obtained using a microfluidizer and had a z-diameter lower than 0.2 µm, while the encapsulation efficiency was not reported [72]. During storage at 25 °C for 50 d, SL nanoparticles emulsified with Quillaja saponin and with Quillaia saponin added to high melting point lecithin retained their α-subcell crystals and provided more than 60% β-carotene retention, while SL nanoparticles made with Quillaia saponin added to low melting point lecithin changed the β-subcell crystals and provided only 20% β-carotene retention (k_25°C_ not reported, Table 1). It was discussed that Quillaia saponin molecules formed a solid two-dimensional adsorption layer with a dense molecule packing because of the formation of multiple hydrogen bonds that inhibited lipid recrystallization. Similarly, high melting point lecithin formed a rigid barrier that inhibited lipid recrystallization in SL nanoparticles [72].

To inhibit bioactive compound expulsion from SL nanoparticles due to recrystallization, one proposed solution was to use a core made of a blend of lipids with different melting points as a strategy to increase the structural disorder. In this line, the formulation of SL nanoparticles with hydrogenated coconut oil or cocoa butter was proposed. In this context, β-carotene was encapsulated in an O/W emulsion made with liquid oils (corn or olive oil) or in SL nanoparticles using high melting temperature lipids (cocoa butter and hydrogenated coconut oil) via cold or hot homogenization respectively, using Tween 80 as a surfactant. These systems were compared for their ability to protect β-carotene, but the encapsulation efficiency was not reported [73]. Oil droplets/nanoparticles with an average diameter of 400, 120, and 200 nm were obtained for olive oil, corn oil, and solid fat, respectively. The ζ-potential was approximately −25 mV for the oil systems and −33 and −39 mV for hydrogenated coconut oil and cocoa butter, respectively. β-carotene stability was found to be at a maximum in the system containing cocoa butter, more than in the other systems. Indeed, the half-life of β-carotene at 25 °C was 40 d, while it was only 10 d in the other systems (k_25°C_ not reported). As expected, liquid oils provided lower protection than the solid fat of cocoa butter, but hydrogenated coconut oil increased the stability with respect to liquid oils. This result was expected, since cocoa butter has a 55% solid fat content at 25 °C, while hydrogenated coconut oil has a 35% solid fat content; thus, the latter can potentially protect β-carotene through a diminished diffusion between the lipid core and the aqueous phase. Moreover, cocoa butter contains longer chain fatty acids with respect to coconut oil, which leads to the formation of polymorphic crystals that are less compact and allow β-carotene to be accommodated inside the structure [73].

To overcome the problem of bioactive compound expulsion from SL nanoparticles, nanostructured lipid (NL) carriers were introduced as a new generation of lipid nanoparticles by blending solid lipids with liquid lipids in order to create a less ordered inner structure [74]. In line with this approach, particles encapsulating β-carotene were obtained via high pressure homogenization and microfluidification, using a medium-chain triglyceride added to increasing amounts of glyceryl stearate. Particles with an average diameter of 96 nm were obtained. β-carotene’s half-life at 25 °C and pH 7.0 increased from 4 d to 10 d (k_25°C_ 0.170 and 0.071 d^−1^, Table 1) with increasing glyceryl stearate content from 0 to 2% [75]. The stabilizing effect of encapsulation was lower than that observed in a previous study [73], probably due to the lower size of the particles. One point to notice is that, compared to O/W emulsions, the SL nanoparticles showed a much lower activation energy, equal to 20 kJ/mol (Table 3) [75]. Hence, O/W emulsions and SL nanoparticles showed different responsiveness to temperature fluctuations and, hence, thermal behavior is another parameter to be considered in order to optimize β-carotene stability.

### 3.6. Rate of Encapsulated β-Carotene Degradation in Oil-in-Water, Oleogel-in-Water, and Oil-in-Gel Pickering Emulsions

A different generation of emulsions was first proposed by Pickering, and these are referred to as “Pickering emulsions” [76] (Table 2). O/W Pickering emulsions are characterized by a colloidal surfactant that provides additional stability to encapsulated compounds and superior stability against coalescence [77]. In one approach, wheat gluten nanoparticles were prepared using a pH-cycle method and then homogenized with β-carotene dissolved in corn oil. Moreover, the resulting Pickering emulsion was homogenized with xanthan gum to obtain a second Pickering emulsion stabilized by gluten nanoparticle–xanthan gum complexes. The gluten nanoparticle–xanthan gum emulsions had larger initial mean particle diameters (23.9 μm) than the gluten nanoparticle ones (9.4 μm). Moreover, the addition of xanthan gum to the wheat gluten nanoparticle Pickering emulsions resulted in the ζ-potential changing from +22.7 to −31.0. However, both emulsions were stable to aggregation over a wide range of pH values (4–8) and salt levels (0–1000 mM NaCl). The retention of β-carotene after thermal treatment at 65 °C for 30 min and 90 °C for 3 min was higher than 97%. The emulsions were also equally efficient in protecting β-carotene during storage, with retentions of around 94.3% and 70.1% after one-month storage at 25 and 37 °C, respectively (k_25°C_ not reported, Table 1) [77]. β-carotene-loaded Pickering emulsions were also obtained using spherical hydrophobic zein colloidal particles and rod-shaped hydrophilic cellulose nanocrystals [78]. The Pickering emulsion solely stabilized by zein colloidal particles showed the largest droplet size, equal to 5.11 µm, while the droplet size of the cellulose nanocrystal-stabilized Pickering emulsion was 3.14 μm, and that of the combined zein and nanocellulose particles at 1:4 ratio was 3.7 μm. The ζ-potential was +17 mV for the emulsions stabilized with zein nanoparticles, −42 mV for the emulsion stabilized with cellulose nanocrystals, and –47 for those stabilized by the combined zein and nanocellulose particles at a 1:4 ratio. Hence, the combination of nanoparticles represents an approach to vary the surface charge and dimension of the particles. Regarding stability, maximum β-carotene retention upon storage at 55°C was observed for the combined zein and nanocellulose particles at a 1:4 ratio, with β-carotene’s half-life higher than 28 d (k_55°C_ not reported, Table 1) [78].

An advanced approach in the design of Pickering emulsion is to use an oleogel as a core, i.e., a three-dimensional gel system that entraps a large volume of liquid oils, thus providing additional stability. In fact, oleogel had previously been found to stabilize oxygen-sensitive compounds [79,80]. To this aim, carboxymethylcellulose was employed to stabilize both an O/W emulsion made via high pressure homogenization followed by sonication, using a soybean oil and oleogel/W emulsion made with a soybean oil structured with beeswax, and these systems were applied to encapsulate β-carotene. The recovery of β-carotene after storage at 25 °C for 15 d was 71.16% and 90.12% in the O/W and oleogel/W Pickering emulsions, respectively. Supposing a first-order kinetics for β-carotene degradation, the expected half-life of this compound at 25 °C would be 31 and 100 d for the O/W and oleogel/W emulsions, respectively (k_25°C_ not reported, Table 1) [81]. However, these data were extrapolated from the degradation curve built for 15 d only and, hence, should be validated.

The stability of Pickering emulsions can also be enhanced by the formation of a three-dimensional viscoelastic network of colloidal particles in the continuous phase. The presence of this 3D viscoelastic particle network enhances emulsion stability by preventing the oil droplets from moving [82]. In this line, gliadin, which can undergo a self-assembly process in water due to its hydrophobicity, was applied to form nanoparticles through the antisolvent procedure [83]. A dispersion of gliadin nanoparticles was then homogenized with β-carotene dissolved in corn oil to form the Pickering emulsion. The percent of gliadin nanoparticles affected the state of the emulsion, which varied form liquid at 0.5% gliadin nanoparticle content to gel-like when the concentration of gliadin nanoparticles was more than 0.75%. The volume-weighted mean diameter was decreased from 11.3 to 4.9 µm by increasing the gliadin nanoparticle concentration from 0.5% to 1.5%. All these systems displayed a β-carotene retention higher than 92% when heated at 70 °C for 30 min or 90 °C for 3 min. The storage stability of these systems was not modeled, but it can be derived that the half-life was higher than 28 d even at 55 °C (Table 1) [84].

A Pickering emulsion containing β-carotene was also developed at a nano-scale. To this aim, chaperonin GroEL, which has a naturally evolved hydrophobic binding rim, was used as an emulsifier. A solution of β-carotene dissolved in rosemary oil was homogenized and ultra-sonicated with a buffered solution of chaperonin GroEL. Overall, the Pickering emulsions with stable nano-droplets of 200−400 nm and a ζ-potential of about −30 mV were formed. During storage at 25 °C, β-carotene retention increased with increasing protein concentrations in the system, with a half-life higher than 35 d (k_25°C_ not reported, Table 1) [85].

**Table 1:** Encapsulation systems systems for the delivery of β-carotene in aqueous environments: matrix ingredient and structure, particle size (PS, µm), ζ-potential (ζ-pot, mV), first-order degradation rate constants (k × 10^3**,**^ d^−1^) and half-life (t**_1/2_**, d) for β-carotene degradation during storage in the dark at a given temperature (T, °C).

Matrix Ingredients	Matrix Structure	PS	ζ-Pot	T	k × 10^3^	t_1/2_	Ref.
carrot	pasteurized juice (90 °C, 60 s)	n.d.	n.d.	4	21	33	[34]
	pasteurized juice (6 μs at 200 Hz, 35 kV cm^−1^ for 1500 μs)	n.d.	n.d.	4	18	39	
carrot	pasteurized juice (180 MPa, 1 pass and 60 °C)	68	−31	4	18	39	[38]
β-carotene, chitooligosaccharides	molecular complexes	0.079	16.5	24	24	28	[43]
β-carotene, zein	molecular complexes	~0.4	−32	20	67	10	[40]
β-carotene, zein, carboxymethylchitosan	molecular complexes	~0.1	−34	20	48	14	
β-carotene, zein, carboxymethylchitosan, tea polyphenols	molecular complexes	~0.08	−40	20	18	39	
β-carotene, yolk lecithin, cholesterol	liposomes	0.247	−25	25	n.d.	6	[48]
β-carotene, yolk lecithin, cholesterol, ascorbic acid	liposomes	0.253	−26	25	n.d.	>30	
β-carotene, medium-chain triglyceride oil, gum arabic	O/W emulsion	n.d.	n.d.	25	95.5	7.2	[49]
β-carotene, medium-chain triglyceride oil, gum arabic, ascorbyl palmitate	O/W emulsion	0.614	−23.8	25	13.9	50	
β-carotene, medium-chain triglyceride oil, gum arabic, α-tocopherol	O/W emulsion	0.644	−26.6	25	14.3	48	
β-carotene, medium-chain triglyceride oil, whey protein	O/W emulsion	0.300	−15	55	n.d.	3	[53]
β-carotene, medium-chain triglyceride oil, whey protein, flaxseed gum	LBL O/W emulsion	0.300	−15	55	n.d.	6	
β-carotene, corn oil, ferulic acid-conjugated curdlan	O/W emulsion	3.62	−30	25	n.d.	7	[55]
β-carotene, sunflower oil, tartary buckwheat bran protein	O/W emulsion	0.959	−8.47	55	n.d.	~7	[56]
β-carotene, sunflower oil, non-covalent tartary buckwheat bran protein–rutin complex	O/W emulsion	0.265	−28.7	55	n.d.	~14	
β-carotene, sunflower oil, covalent tartary buckwheat bran protein–rutin complex	O/W emulsion	0.243	−32.7	55	n.d.	>>28	
β-carotene, camellia oil, low-methoxy pectin	O/W emulsion	20	~22	4	n.d.	~4	[57]
β-carotene, camellia oil, covalent low-methoxy pectin/soy peptide complex	O/W emulsion	11	~18	4	n.d.	>>28	
β-carotene, camellia oil, covalent low-methoxy pectin/corn peptide complex	O/W emulsion	17	~20	4	n.d.	~4	
β-carotene, camellia oil, covalent low-methoxy pectin/whey protein peptide complex	O/W emulsion	~14	~ 18	4	n.d.	>>28	
β-carotene, canola oil, Tween 80, alginate	O/W emulsion	n.d.	n.d.	25	n.d.	48	[61]
β-carotene, canola oil, Tween 80, alginate, calcium	O/gel emulsion	n.d.	n.d.	25	n.d.	99	
β-carotene, tristearin, Quillaja saponin	SL nanoparticles	<0.200	n.d.	25	n.d.	>50	[72]
β-carotene, tristearin, Quillaja saponin, high melting point lecithin	SL nanoparticles	<0.200	n.d.	25	n.d.	>50	
β-carotene, tristearin, Quillaja saponin, low melting point lecithin	SL nanoparticles	<0.200	n.d.	25	n.d.	~30	
β-carotene, olive oil, Tween 80	O/W emulsion	~0.400	−28	25	n.d.	~10	[74].
β-carotene, corn oil, Tween 80	O/W emulsion	~0.120	−25	25	n.d.	~10	
β-carotene, cocoa butter, Tween 80	SL nanoparticles	~0.200	−39	25	n.d.	~40	
β-carotene, hydrogenated coconut oil, Tween 80	SL nanoparticles	~0.200	−33	25	n.d.	~10	
β-carotene, medium-chain tryglicerides, Tween 80	O/W emulsion	0.106	~−30	25	170	4	[75]
β-carotene, medium-chain triglycerides, 2% glyceryl stearate Tween 80	NL carriers	0.096	~−30	25	71	10	
β-carotene, corn oil, gluten	O/W Pck emulsion	9.4	+22	25	n.d.	>>30	[77]
β-carotene, corn oil, gluten, xanthan gum	O/W Pck emulsion	23.9	−31	25	n.d.	>>30	
β-carotene, sunflower oil, medium-chain triglycerides, cellulose nanocrystals	O/W Pck emulsion	3.14	~−42	55	n.d.	~5	[78]
β-carotene, sunflower oil, medium-chain triglycerides, zein nanoparticles	O/W Pck emulsion	5.11	~ +17	55	n.d.	>28	
β-carotene, sunflower oil, medium-chain triglycerides, 1/4 (*w*/*w*) zein nanoparticles/cellulose nanocrystals	O/W Pck emulsion	~3.7	~−47	55	n.d.	>28	
β-carotene, soybean oil, cellulose nanocrystals	O/W Pck emulsion	8.34	n.d.	25	n.d.	30	[81]
β-carotene, soybean oil, beeswax, cellulose nanocrystals	Ogel/W Pck emulsion	20.16	n.d.	25	n.d.	100	
β-carotene, corn oil, gliadin	O/gel Pck emulsion	4.9	n.d.	55	n.d.	>28	[84]
β-carotene, rosemary oil, chaperonin GroEL	O/W Pck emulsion	~0.40	−30	25	n.d.	>35	[85].

Rate constants and half-lives refer all-*trans*-β-carotene degradation. O, oil; W, water; SL, solid lipid; LBL, layer by layer; NL, nanostructured lipid; Pck, Pickering; Ogel, oleogel; n.d., not determined.

**Table 2:** Micro- and nano-structures discussed in the text and related strategies to decrease the rate of β-carotene degradation. O, oil; W, water; LBL, layer by layer; SL, solid lipid; Pck, Pickering; Ogel, oleogel.

Structure	Strategies to Increase β-Carotene Stability
molecular complexes 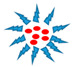	Use of carrier biopolymers with antioxidant activity to inhibit radical-mediated oxidation
liposomes 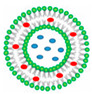	Co-encapsulation of antioxidant compounds to inhibit radical-mediated oxidation
O/W emulsion 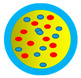	Functionalization of the emulsifier to increase its oxygen barrier properties
LBL O/W emulsion 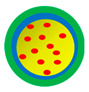	Addition of multiple biopolymer layers to decrease oxygen diffusion rate at the O/W interface
O/gel emulsions 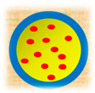	Use of a gelled continuous phase to decrease oxygen diffusion rate at the air/continuous phase interface and at the continuous phase/oil droplet interface
SL nanoparticles 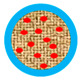	Use of a solid lipid phase to decrease oxygen diffusion rate through the core
O/W Pck emulsions 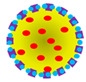	Use of colloidal surfactants to decrease oxygen diffusion rate at the O/W interface
Ogel/W Pck emulsions 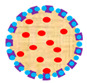	Use of colloidal surfactants and an Ogel as a core to decrease oxygen diffusion rate at the Ogel/W interface and through the core
O/gel Pck emulsions 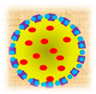	Use of colloidal surfactants and a gelled continuous phase to decrease oxygen diffusion rate at the air/continuous phase interface and at the continuous phase/oil droplet interface

**Table 3:** Activation energy (Ea, kJ/mol) in a given temperature range (T range, °C) for the degradation of β-carotene encapsulated in nano-and micro-structures in aqueous environments.

Matrix Ingredients	Matrix Structure	T Range	Ea	Ref.
β-carotene, zein	molecular complexes	4–60	13 (calculated)	[40]
β-carotene, zein, carboxymethyl chitosan	molecular complexes	4–60	7 (calculated)	
β-carotene, zein, carboxymethyl chitosan, tea polyphenols	molecular complexes	4–60	17 (calculated)	
β-carotene, medium-chain triglyceride oil, gum arabic, α-tocopherol	O/W emulsion	4–65	49.8	[49]
β-carotene, medium-chain triglyceride oil, gum arabic, ascorbyl palmitate	O/W emulsion	4–65	45.7	
β-carotene, canola oil, Tween 80, alginate	O/W emulsion	4–37	51.1	[61]
β-carotene, canola oil, Tween 80, alginate, calcium	O/gel emulsion	4–37	47.6	
β-carotene, medium-chain tryglicerides, Tween 80	O/W emulsion	4–35	58 (calculated)	[75]
β-carotene, medium chain tryglicerides, glyceryl stearate, Tween 80	SL nanoparticles	4–35	20 (calculated)	

Activation energy refers to all-*trans*-β-carotene degradation. O, oil; W, water; SL, solid lipid.

## 4. Conclusions and Future Perspectives

In liquid systems, such as carrot juice, β-carotene stability is relatively low, with a half-life of about 30 d at 4 °C. Most of the nano- and micro-structures designed for β-carotene encapsulation and dispersion in the water phase provided better protection, since a similar half-life was found upon storage at room temperature. Improved β-carotene stability was achieved by using emulsifiers or wall materials with antioxidant activity or upon incorporation in the structure of lipid-soluble antioxidants, such as ascorbyl palmitate and α-tocopherol, which can scavenge oxygen radicals and decrease the rate of the oxidative process, with an increase in the half-life of about two times. Nevertheless, the most promising approach to increase β-carotene stability was found to be the design of heterogeneous structures where the core is partially or fully solidified, or either the core or the surface or the outer phase are gelled. In these latter structures, the half-life of β-carotene was found to increase to 100 d at room temperature or more, most likely because the diffusion processes were limited due to the increased viscosity. In this context, the study of oxygen diffusion rates within various matrices would support the rational design of encapsulated structures, since oxidation is the leading cause of β-carotene loss. Moreover, knowledge on the physical and chemical stability of the encapsulated systems under a wide pH range, ionic strength, and processing temperature should be extended to assist their application in foods. One aspect to underline is that the kinetic data collected in this study were obtained from model systems built with refined compounds in order to obtain insight into the role of specific molecular components. The main challenges in future studies will be to further improve process sustainability by using unrefined β-carotene sources and/or surfactants and/or wall materials. Finally, considering the importance of the gelled phase for β-carotene stability, the extraordinary array of biopolymers available in nature could be explored to develop the formulations that better combine process sustainability, β-carotene stability, and bioavailability.
